# The IDEAL framework in neurosurgery: a bibliometric analysis

**DOI:** 10.1007/s00701-020-04477-5

**Published:** 2020-07-10

**Authors:** Helen C. U. Ota, Brandon G. Smith, Alexander Alamri, Faith C. Robertson, Hani Marcus, Allison Hirst, Marike Broekman, Peter Hutchinson, Peter McCulloch, Angelos Kolias

**Affiliations:** 1 St Mary’s Hospital, London, UK; 2 Department of Clinical Neurosciences, University of Cambridge & Addenbrooke’s Hospital, Cambridge, UK; 3 Department of Neurosurgery, The Royal London Hospital, London, UK; 4 Department. of Neurosurgery, Massachusetts General Hospital, Boston, MA USA; 5 The Victor Horsley Department of Neurosurgery, The National Hospital for Neurology and Neurosurgery, London, UK; 6 IDEAL Collaboration, Nuffield Department of Surgical Sciences, University of Oxford and John Radcliffe Hospital, Oxford, UK; 7 Department of Neurosurgery, Haaglanden Medical Center/Leiden University Medical Center, The Hague, Netherlands

**Keywords:** Research, Evidence, Innovation, Neurosurgery, Surgery, IDEAL framework

## Abstract

**Background:**

The Idea, Development, Exploration, Assessment and Long-term study (IDEAL) framework was created to provide a structured way for assessing and evaluating novel surgical techniques and devices.

**Objectives:**

The aim of this paper was to investigate the utilization of the IDEAL framework within neurosurgery, and to identify factors influencing implementation.

**Methods:**

A bibliometric analysis of the 7 key IDEAL papers on Scopus, PubMed, Embase, Web of Science, and Google Scholar databases (2009–2019) was performed. A second journal-specific search then identified additional papers citing the IDEAL framework. Publications identified were screened by two independent reviewers to select neurosurgery-specific articles.

**Results:**

The citation search identified 1336 articles. The journal search identified another 16 articles. Following deduplication and review, 51 relevant articles remained; 14 primary papers (27%) and 37 secondary papers (73%). Of the primary papers, 5 (36%) papers applied the IDEAL framework to their research correctly; two were aligned to the pre-IDEAL stage, one to the Idea and Development stages, and two to the Exploration stage. Of the secondary papers, 21 (57%) explicitly discussed the IDEAL framework. Eighteen (86%) of these were supportive of implementing the framework, while one was not, and two were neutral.

**Conclusion:**

The adoption of the IDEAL framework in neurosurgery has been slow, particularly for early-stage neurosurgical techniques and inventions. However, the largely positive reviews in secondary literature suggest potential for increased use that may be achieved with education and publicity.

## Introduction

With technological advances leading to rapid development of new devices and operative techniques, it remains imperative that we critically assess novel ideas to ensure they confer true patient benefit. Innovative solutions to unique problems are reliant on creativity and lateral thinking, which can appear at odds with the rules and regulations required to systematically appraise developments. However, it is in the interest of patient safety and the society to ensure that widespread implementation occurs after rigorous assessment and research [18].

The IDEAL framework is a straightforward and structured approach that can guide evaluation and research across all surgical fields, while also allowing enough flexibility to prevent stifling of innovation. First published in 2009 [41], it was developed to provide guidance on the evaluation of surgical techniques and devices from inception to long-term evaluation [7]. It consists of 5 stages: Idea, Development, Exploration, Assessment, and Long-term studies. Each stage includes various recommendations to ensure that high-quality evidence is obtained when studying new ideas, as demonstrated in Table 1.

**Table 1:** Table outlining the IDEAL framework recommendations (adapted with permission from the authors)[25]

	1 Idea	2a Development	2b Exploration	3 Assessment	4 Long-term study
Purpose	Proof of concept	Development	Learning	Assessment	Surveillance
Number and types of patients	Single digit; highly selected	Few; selected	Many; may expand to mixed; broadening indication	Many; expanded indications (well defined)	All eligible
Number and types of surgeons	Very few; innovators	Few; innovators and some early adopters	Many; innovators, early adopters, early majority	Many; early majority	All eligible
Output	Description	Description	Measurement; compassion	Comparison; complete information for non-RCT participants	Description; audit, regional variation; quality assurance; risk adjustment
Intervention	Evolving; procedure inception	Evolving; procedure development	Evolving; procedure refinement, community learning	Stable	Stable
Method	Structured case reports	Prospective development studies	Research database; explanatory or feasibility RCT (efficacy trial); diseased based (diagnostic)	RCT with or without additions/modifications; alternative designs	Registry; routine database
Outcomes	Proof of concept; technical achievement; disasters; dramatic successes	Mainly safety; technical and procedural success	Safety; clinical outcomes (specific and graded); short-term outcomes; patient-centred (reported) outcomes; feasibility outcomes	Clinical outcomes (specific and graded); middle-term and long-term outcomes; patient-centered (reported) outcomes; cost-effectiveness	Rare events; long-term outcomes; quality assurance
Ethical approval	Sometimes	Yes	Yes	Yes	No

This framework differs from the traditional model of pharmacological trials to accommodate the distinct way surgical developments occur, particularly since individual operator skill and technique modifications at the early stages can differentially impact outcomes.[46]. A subsequent paper introduced Stage 0, a preclinical stage, for testing involving cadavers, animals, or simulations, while another clarified the use of the IDEAL framework in surgical device development [25, 56]. The IDEAL structure adds value to surgical innovation by ensuring safety and regulation; however, uptake has varied across various countries and surgical specialties. While utilization has increased overall, this has predominantly happened in other surgical fields [28]. A recent review of the IDEAL framework applied within minimally invasive neurosurgical research assessed historical studies involved in the development of the endoscopic endonasal approach for skull base meningiomas and the Woven EndoBridge (WEB) device for endovascular treatment of intracranial aneurysms [48]. The authors assessed the quality of the research that contributed to these developments by measuring the adherence of various studies to IDEAL guidelines. Their search revealed a total of four clinical papers that could be aligned with any stage of the IDEAL framework.

The aim of this bibliometric review is to investigate the adoption of the IDEAL framework in neurosurgical literature as a whole and reflect on factors influencing implementation since its inception 10 years ago.

## Methodology

We performed individual citation searches for the seven main IDEAL papers [13, 18, 19, 25, 41, 42, 56] on Scopus, PubMed, Embase, Web of Science, and Google Scholar databases (September 2009 to August 2019). Articles were identified and analyzed by two independent reviewers, CO and BS. These searches were completed on each database individually. Papers identified were stored and deduplicated in Mendeley reference manager. The titles and abstracts of all papers were then screened for relevance to the study by the two independent reviewers, followed by full text review; the final selection was reviewed by a third independent reviewer to ensure suitability for inclusion (AA). Any disagreements were resolved following discussion with the senior author (AK). All papers with a focus on neurosurgical techniques or devices published between September 2009 and August 2019 were included. Non-English papers and non-journal text (such as book chapters) were excluded. This methodology was first used by Tradewell et al., investigating the use of the IDEAL framework in urological research [57].

For completeness, a second search using the terms “IDEAL Collaboration” and “IDEAL framework” was performed in the following major neurosurgical journals: *Acta Neurochirurgica, British Journal of Neurosurgery*, *Child’s Nervous System*, *Clinical Neurology and Neurosurgery*, *Journal of Clinical Neuroscience*, *Journal of Korean Neurosurgical Society*, *Journal of Neurology*, *Neurosurgery and Psychiatry*, *Journal of Neurosurgery*, *Journal of Neurosurgery*: *Pediatrics*, *Journal of Neurosurgery*: *Spine*, *Journal of Neurosurgical Sciences*, *Neurosurgery*, *Neurosurgery Clinics of North America and Clinical Neurosurgery*, *Neurosurgical Focus*, *Neurosurgical Review*, *Pediatric Neurosurgery*, *Stereotactic and Functional Neurosurgery*, *Turkish Neurosurgery*, and *World Neurosurgery.*

Primary papers were defined as original research publications assessing new surgical techniques or devices, and were linked to the most relevant stage of the IDEAL protocol (many self-identified) and assessed for adherence to criteria for that stage. Secondary papers included systematic reviews, opinion pieces, and letters to the editor; these were assessed with regard to their support for and evaluation of the IDEAL papers and framework.

## Results

**Fig. 1:**
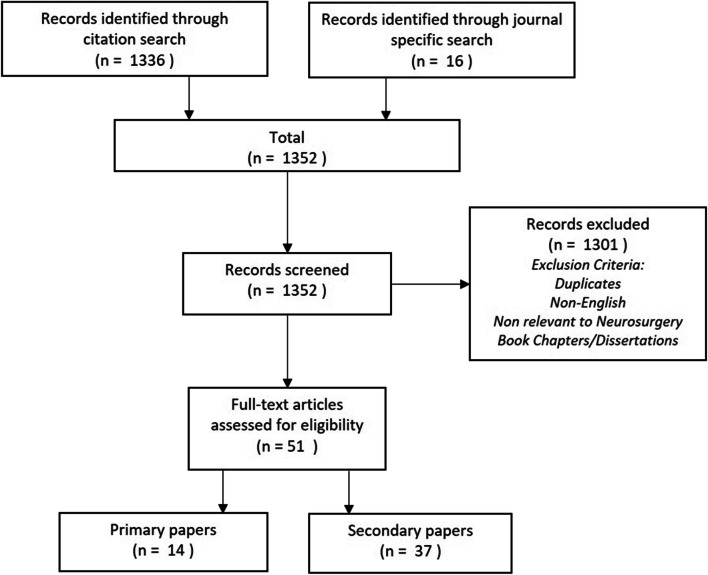
Diagram outlining search strategy

The citation search identified 1336 articles, while the secondary search identified another 16 (see Fig. 1). Following deduplication and abstract review, 51 relevant articles were identified. Thereafter remained fourteen primary papers [8, 17, 20, 26, 33, 34, 37, 39, 46, 51, 55, 58–60], and 37 secondary papers [1–5, 9–12, 14–16, 23, 27, 29–32, 35, 36, 38, 40, 43–45, 47–50, 52–54, 57, 62, 64–66].

Articles were published from 2010 to July 2019 (date of citation search), with a peak in 2018. The top 3 journals for publications that cited the IDEAL articles were *Acta Neurochirurgica* (*n = 8*), *Journal of Neurosurgery* (*n = 5*), and *World Neurosurgery* (*n = 5*)*.*

### Primary publication analysis

All of the fourteen primary publications identified were from European groups. Papers were published between 2013 and 2019 and spanned a number of sub-specialties including skull base, neuro-oncology, and spinal neurosurgery. Ten papers (71%) explicitly mention the IDEAL framework within their text [8, 20, 26, 33, 34, 37, 39, 55, 58, 60]. Six (43%) papers aimed to align their research with a specific stage of the IDEAL framework [8, 20, 33, 34, 39, 60]. Of these six, five (83%) papers appropriately applied the IDEAL framework to their research; one publication claiming to align to stage 1/2a was an animal study [8]. Animal studies are technically stage 0, the preclinical stage of the IDEAL framework [33].

Of the five papers appropriately staged, their adherence to IDEAL recommendation including study size, ethical oversight, and outcome measures are presented in Table 2*.*

**Table 2:** Publication adherence to IDEAL framework (N/A refers to criteria not necessary to fulfill a specific stage of the IDEAL framework)

	Belotti et al.[8]	Versteeg et al.[60]	Majovsky et al.[34]	Flores et al.[20]	Marcus et al.[39]
IDEAL stage	0	1/2a	2a	2a	0 (Ideal-D)
Study Size (Mean)	10 specimen	3 (Stage 1), 10 (Stage 2a)	18	30	15
Follow up time (months, mean)	N/A	13	9.3	6.5	N/A
Number of surgeons reported (number of surgeons)	No (N/A)	No (N/A)	No (N/A)	No (N/A)	Yes (4)
Number of clinical sites reported (mean number of clinical sites)	Yes (2)	Yes (1)	No (N/A)	No (N/A)	Yes (1)
Prospective study	N/A	Yes	Yes	No	N/A
Prior published/registered protocol	N/A	N/A	No	No	N/A
Safety/feasibility of procedure considered	Yes	N/A	N/A	Yes	Yes
Ethical oversight	N/A	Yes	No	No	N/A
Document adverse outcomes	N/A	Yes	Yes	Yes	N/A
Sequential reporting of outcomes	N/A	Yes	Yes	Yes	N/A
Patient reported outcomes	N/A	No	Yes	Yes	N/A
Participants registered in a database	N/A	No	No	No	N/A
Surgeons view of procedures considered	N/A	No	N/A	No	N/A
Documented funding	No	Yes	Yes	No	Yes

The paper by Belotti et al. aligned with Stage 0, the preclinical stage of the IDEAL framework [8]. This study involved the use of 10 specimens for testing in one clinical center, another two for filming in a second center. The purpose of the study was to improve safety and effectiveness of the transsphenoidal approach by categorizing different approaches, aligning with Stage 0. Marcus et al. also aligned their study to Stage 0, within the IDEAL-D framework specific to devices; comparing the use of computer-assisted planning with manual planning in stereotactic brain biopsy [39]. The study by Versteeg et al. that investigated spinal stabilization following radiotherapy had two arms. Each arm was applied appropriately to the Idea (1) and Development (2a) stages with samples sizes of 3 and 10, respectively [60]. Adverse events were evaluated and classified according to the common toxicity criteria adverse events during a median clinical follow-up time of 13 months. The study by Majovsky et al. was stage 2a and assessed the use of burr holes in evacuation of chronic subdural hematomas [34]. The paper by Flores et al. was also aligned to stage 2a, investigating the use of syringes as dilators and retractors in spinal surgery in 30 patients [20]. None of the studies included from the citation search identified as stage 2b, 3, or 4 (exploration, assessment, long-term monitoring).

### Secondary publication analysis

Thirty-seven secondary papers were identified. Of them, 13 (35%) were narrative reviews [9, 11, 15, 27, 29–31, 43–45, 52, 53, 64]. Eleven (30%) were systematic reviews [1, 5, 10, 14, 16, 38, 40, 46, 48, 49, 66].

The remaining papers identified are consist of 10 letters to the editor (27%) [2–4, 12, 23, 32, 36, 47, 50, 54], 2 questionnaire-based analyses (one exploring views on the use of evidence-based medicine principles in neurosurgery, the other seeking to understand the neurosurgical definition of “innovation”) [62, 65], and 1 article outlining the work of the British Neurosurgical Trainee Collaborative [57].

Of the secondary publications, 21 (57%) explicitly mentioned the IDEAL framework [1, 3, 5, 10–12, 14, 15, 29, 32, 38, 43, 47–50, 52, 53, 57, 60, 65], while the rest cited the IDEAL publications, but did not discuss them. Of the 21 papers that discussed the IDEAL framework, 18 (86%) were supportive of the use of the IDEAL framework [3, 5, 10–12, 14, 15, 29, 32, 38, 43, 47, 49, 50, 52, 53, 57, 60], while 1 paper was not [1], and 2 were neutral [48, 65]. Six of these publications evaluated the applicability of the IDEAL framework in neurosurgical innovation [38, 47, 48, 50, 64, 65].

## Discussion

The aim of this study was to explore the adoption of the IDEAL framework within neurosurgery by reviewing citations of the key IDEAL papers. The citation search allowed us to assess awareness of the framework, and examine the way in which it is utilized. This methodology was informed by the work of Tradewell et al. who also used a citation search to review the use of the IDEAL framework in urological literature [21]. Given the large number of citations of these papers in urological literature, it is clear that the framework has impacted thinking in surgical development; however, this does not seem to be reflected particularly in neurosurgery, evident by the small number of papers that specifically cited the framework, or were aligned to a specific stage of the framework. Encouragingly, the number of neurosurgical papers referencing the framework has shown a year on year increase; however, numbers remain relatively small.

There are several factors that could contribute to the limited uptake of the IDEAL framework in neurosurgery. A potential cause is a lack of awareness. The IDEAL framework was developed in the UK, and initially published in 2009 [41]. Though there are a number of articles that outline the IDEAL framework and its recommendations, it is possible that those involved in current research and surgical development are either not aware, or not particularly well versed in the framework. This is supported by our finding that all primary papers were of European origin. It is also important to consider that many researchers may not have considered the need for a framework specifically designed for surgical innovation, and therefore did not seek one out. Given the low numbers of primary papers that cited and applied the IDEAL framework to their research, and given that some of these papers did so incorrectly or incompletely, it is clear that education on the IDEAL framework is still actively required to guide researchers and authors, a problem also identified by Khachane et al. [28]. The IDEAL council has identified this potential issue, and encourages surgeons from all specialties and any country to join the collaboration, particularly through the use of a diverse group of council advisors and specialty leads from a number of different countries and specialties, who help to develop and promote the IDEAL framework. The IDEAL council view the framework as ever-evolving in response to pragmatic need and a requirement to learn from IDEAL’s end-users. The update paper was also published in the Annals of Surgery, a US Journal, to further increase the reach of the framework [25].

It is also important to consider how applicable the framework is to neurosurgical research. Surgical innovation and technical development differ between specialties and even sub-specialties, as illustrated in Muskens et al.’s examination of innovation within endoscopic endonasal surgery for skull base meningiomas versus the Woven EndoBridge device for endovascular treatment of intracranial aneurysms. When they explored mapping those developments to the IDEAL framework, only four of the 21 papers included could be matched to any stage of the IDEAL framework: two papers to Stage 2A and two to 2B [48]. Looking specifically at the lack of papers that align with stage 2B, as replicated by our own results, a potential reason for the low numbers of this type of study is that neurosurgical research still largely follows the traditional model of a phase 2 randomized trial that may or may not be followed by a phase 3 randomized trial. A recent example is the MISTIE II and MISTIE III trials (phase 2 and phase 3 trials), which evaluated a new technique for evacuation of intracerebral hemorrhage (ICH) [22, 63]. Given these studies fulfill much of the criteria for IDEAL stages 2b and 3, it is clear that potential to align these studies is the IDEAL framework is present; however, tradition or simply lack of awareness of the IDEAL framework may have prevented this.

Another potential reason is that, as is often the case with surgical research, there is an overreliance on retrospective “case series.” This is likely because case series are easy to perform, require less resources, can be conducted at a single center, and, for many surgeons, are means to showcase their surgical skills and outcomes [61]. Furthermore these studies afford a flexibility that may not be afforded by adhering strictly to the IDEAL framework. Some surgeons would argue that limiting this flexibility stifles innovation. However, the true aim of the framework is to facilitate the conduct of well-designed and well-executed studies in order to facilitate the adoption of innovative techniques, if found to be effective. A recent example is the use of middle meningeal artery (MMA) embolization for chronic subdural hematomas (CSDH), where the majority of conducted studies are case series [6]. Although this procedure is clearly innovative, many of the studies reported use of MMA embolization for atypical indications (e.g., asymptomatic patients, as “prophylaxis” after surgery) outside the context of ethically approved research.

The lack of IDEAL stage 3 studies, which are typically a definitive, multi-center randomized trial, can be explained by the fact that these studies are usually identified as phase 3 trials or simply randomized trials, despite technically meeting the criteria of a stage 3 IDEAL study. A recent review identified 401 published randomized trials on brain and spine conditions treated by neurosurgeons from 2003 to 2016 [24]. Given these numbers, it appears that randomized trials are possible within neurosurgical innovation, and therefore the potential for stage 2b and stage 3 IDEAL studies is much greater than what is currently present. In order to investigate this further, qualitative feedback should be sought from neurosurgeons on the applicability of the IDEAL framework to their research, in order to identify factors that have limited or prevented use. This information may in turn be used to guide future updates of the framework, and educational materials used within the neurosurgical community.

As most developments occur in incremental improvements upon techniques or devices, the appropriateness of large randomized controlled trials for each small, additive change is questionable. However, this would not apply to innovations, such as MMA embolization or the MISTIE procedure, which are entirely different to the usual method of treating CSDH and ICH, respectively [21, 22, 61]. There are also ethical considerations; if a new technique or surgical device displays a substantial, unequivocal benefit over past standard (e.g. the introduction of microscope in micro-neurosurgery), the lack of clinical equipoise precludes conducting a trial that may expose patients to suboptimal treatment.

There are also ongoing concerns that neurosurgical randomized trials are often not feasible or impractical [64]. Some authors suggest that routinely collected or observational data can lead to robust conclusions regarding the comparative effectiveness of treatment; however, this is a relatively new field with ongoing methodological challenges. As an example, a recent study found that observational studies based on routinely collected health data could give different answers from subsequent randomized trials on the same clinical questions and may substantially overestimate treatment effects [6]. Reasons for this are likely multiple, and while this difference could reflect a difference in validity achieved by the different study methodologies, it is important to consider the impact the highly selective populations used for randomized trials could have on research outcomes, in comparison with the broader populations that usually contribute to observational studies. Another option, which is gaining traction in recent years, is the use of pragmatic large randomized trials that have broad inclusion criteria in order to reflect real-world practice. The IDEAL collaboration is working on developing guidance on the use of real-world evidence for the purposes of comparative effectiveness research.

The most recent IDEAL publication, a follow up to the first paper, has elaborated more on the recommendations and how to apply them for each stage [25]. It is hoped that this new update of the IDEAL framework will improve the understanding of where the framework can fit within neurosurgery and therefore improve uptake. Further projects include stage-specific reporting guidelines developed using Delphi methodology. Given that the majority of secondary papers that cited the IDEAL framework were supportive of its use, it would seem that education, promotion, and room for specialty-specific nuance within recommendations could largely improve uptake, and in turn help guide neurosurgical development to produce a high-quality evidence base for our practice.

### Limitations of this paper

This paper reviewed articles that cited the IDEAL framework key papers. It is possible that there are studies that have adhered to and referenced the framework, but have not cited these papers and are therefore excluded from this review. The secondary journal specific search aimed to negate this limitation; however, this solely identified articles within the selection of journals searched (listed above). Papers published in other journals will not have been identified in this search. It is also possible that there is a research that has unintentionally adhered to the IDEAL framework but has not been included, again as they have not cited the IDEAL papers. Evaluation of the papers and their adherence to the IDEAL framework recommendation (primary papers) or support of the IDEAL framework (secondary papers) was subjective, as based on the opinion and understanding of the authors of this paper.

## Conclusion

Ultimately, in order to fully evaluate the potential for the IDEAL framework in neurosurgical research, it is necessary that more primary research studies attempt to follow the recommendations. Feedback highlighting neurosurgery-specific limitations can be generated and incorporated into future iterations of the framework. This will ensure that it is able to support and work with nuances and specialty-specific concerns that are causing limited use of the IDEAL framework thus far.
